# Supplementary material for: Patterns of Intron Gain and Loss in Fungi
Source: PLoS Biol. 2004 Nov 30;2(12):e422. doi: 10.1371/journal.pbio.0020422 (PMC532390; doi:10.1371/journal.pbio.0020422)
Supplement: Table S1 — Also available at http://genes.mit.edu/NielsenEtAl/. (4.3 MB ZIP). [file pbio.0020422.st001.zip › NielsenEtAl/html/1201.html]

AN6046.1.NCU07850.1.MG05280.1.FG04123.1


```
 CLUSTAL W (1.82) Multiple Sequence Alignments - Introns Inserted


Sequence 1: NCU07850.1	470 aa
Sequence 2: MG05280.1	525 aa
Sequence 3: FG04123.1	509 aa
Sequence 4: AN6046.1	517 aa
Alignment Length: 554 aa
Number Identitical Residues: 182 aa
Alignment Score (without introns) 10016


MG05280.1 	MSLKQ0EIETWVTALGRYDNNEFEDALGEFDKISDTSKILFNMGVIHATLGEHEKA0VEC
NCU07850.1	MSLKQ0EIETWVSALAQYDNSEFDAALQEFEKIADTSKILFNMGVIHATLGQHEQA0VEC
FG04123.1 	MSLKQ0EIETWVAALGRYDNNEFEEALNEFGKIGDTSKILFNMGVIHATLGEHEKA0VES
AN6046.1  	MSLKQ0EIETWVQALEHFDNQEYDLALRSFAAIADTSKILFNCGVIYATLGEHEKA0VEC
          	***** ****** ** ::**.*:: ** .*  *.******** ***:****:**:* **.

MG05280.1 	YQRAIKLDQYLAVAYFQQGVSNFLLGDFEEALANFNDTLLYLRGNTMIDYAQLGLMFKLY
NCU07850.1	YQRAIRLDQYLAVAYFQQGVSNFLLGDFEEALANFNDTLLYLRGNQVIDYAQLGLLFKLY
FG04123.1 	YQRAIRLDQYLAVAYFQQGVSNFLLGDFEEALANFNDTLLYLRGNAMIDYAQLGLLFKLY
AN6046.1  	YQGAVGLDQYLAIAYFQEGVSNFLLGDFEEALANFNDTLLYLRGNTYIDYEQLGLKFRLY
          	** *: ******:****:***************************  *** **** *:**

MG05280.1 	SCEVLFNRGLCYIYLQQKDAGLQDLNYAVKEK~VVEDHNVIDDAIREEAE0GYTVFSIPV
NCU07850.1	SCEVLFNRGLCYIYLQQIEAGMQDLSYAAKEK~VVEDHNVIDEAIKENAE0LLTLG----
FG04123.1 	SCEVLFNRGLCYIYLQQMDAGMQDFSYAVKEK~VVEDHNVIDDAINEQAE0GYTVFSIPV
AN6046.1  	SCEVLFNRGLCYIYLQQIGPGMQDLEYASKEK0YSRSR--------LTPQ~GYTVFSIPV
          	*****************  .*:**:.** ***   ..:          .:   *: : . 

MG05280.1 	GVVYRPNDAKVRNLKTKDYLGKARLVAASDRSNAFTGFAGSEIKN~SGKADVKDDRPSDN
NCU07850.1	-------------------------------------------NQ~AGKLEVKDDRPVES
FG04123.1 	GVVYRPNEAKVRNLKTKDYLGKARLVAASDRANAFTGFAGSEIKN0AGKLEVKDDRPADN
AN6046.1  	GVVYRPNEAKVKNLKTKDYLGKSRVIAA---------------NR~LSTPADTSQRSVDS
          	.    ...:.  . .:..  ..:   ::               :.  ..   ..:*. :.

MG05280.1 	ISFAATNLVKPGLQSRARQQSEPPNSRNVFPPTPPPENDKQTMSRGAS-----VRNGPKP
NCU07850.1	LSFAATNLVKPGLQSR-RQQSEPPTNRSMFPPTPPPENEKSQMSRGAS-----VRNGPKP
FG04123.1 	ISFAATNLVKPGLSSR-RQQSEPPNGRNVFPPTPPPENERP--SRAAS-----VRN-QKP
AN6046.1  	VPFATSHLVQKNLTSRSRQQSEPPMHRNLFPPTPPPDADKASLSSTGSNGTVRAQPGKAQ
          	:.**:::**: .* **:*******  *.:*******: ::   *  .*..:  .: .   

MG05280.1 	MPAKLNIDKARPNDRYERTTSPQEQRSRAPSRAPSNAPSRGFSTRE---PPRRRPRSQE-
NCU07850.1	MPARLNLDKVRPSDRYEKTSP--EEPSRRPSRAASATPSRGFSQREQPPPPRMRRSQQE-
FG04123.1 	QLAKLNIQQAEPNRRYEKAAS--PADARRP-MPRSASTTRTPLQRE-PPPLQLRPKQIP-
AN6046.1  	RPPKLDLDRPGAQPAGRSTTDLTAPEKPRLGTIRTASEPRGQSRQPRGYAPERHVRSSDG
          	  .:*::::  ..   . ::          .   : : .*    :    . . :  .  .

MG05280.1 	-------------EEEGYLDDVYDNYGGGIGGGGGSIRGSRSQRRAPQQRYIEEEDE--G
NCU07850.1	-------------EEDSYPSELYDMYGG--PGPNRNSRGQRSNRSAP--RYIQEEDD--E
FG04123.1 	-------------EETGSPEDVYAMYSA--TDGYRNSRGSAGSRRLRPQQYSEEED---A
AN6046.1  	YGHRRGASDHGFGVSNGHSDDAYGMYGE-----ARAMTLANGGRPFQQQGYIDEEEEYGS
          	 .   .::. . . . .  .: *  *.              . *    . * :**:. . 

MG05280.1 	SDFDDGSFDEGDFEMVSNRRPGTNSMSSSS-RGQSRRPDIRNIKVKVHYTATDDTRMILI
NCU07850.1	SDYD-VSFDEAEFEMVSGRRPSLSTVRSSGGRGSSRRPELRSIRVKVH---AGDVRYIMV
FG04123.1 	SDYE-GTINENDFEMIGQRR-GPGSVSGSR---NSRRTEVTKIRVKVH---ADEVKLIMI
AN6046.1  	SPCDEDLVPDASFELMGSRPRARSCSRGPA-RGYSRRPEVRRFRVKVHS--FEDTRYILI
          	*  :.  . : .**::. *  . .   ..   . ***.::  ::****     :.: *::

MG05280.1 	GMACEYSDFVEKIRNKFKIASRRSVRIKVRDDED-GGTMITMGDQDDLDMAIMASKSMAR
NCU07850.1	GTAIEFPDFEEKIRSKFGIRRR--IKIKIKDDDSPDGDMITVGDQDDLDMLIQTVKQNAR
FG04123.1 	TPDTRFETLSDKVRDKFNIKRR--FKIKVKDDDMPNGDMITVGDQDDLEMVIDSVKDEAR
AN6046.1  	PPTIEFAEFETRIREKFGFQMA--LKIKMQD----EGDMITMVDQEDLDLLLMASREIAR
          	    .:  :  ::*.** :     .:**::*     * ***: **:**:: : : :. **

MG05280.1 	RERSDNGKME0VWIQDA---
NCU07850.1	KQRLETGKME0VWVQEI---
FG04123.1 	KQRTETGKME0IWILQL---
AN6046.1  	REGSEMGKME0IWVEERRMI
          	::  : **** :*: :
```
